# Assessing the determinants and association of cognitive memory performance with blood folate and cobalamin levels in Qatar’s healthy aging population

**DOI:** 10.5339/qmj.2025.109

**Published:** 2025-12-10

**Authors:** Hina Akram, Nasseer Masoodi, Muhammad Abd Ur Rehman, Zumin Shi, Manar E. Abdel-Rahman

**Affiliations:** 1 Department of Public Health, Qatar University, Doha, Qatar; 2 Department of Internal Medicine, Hamad Medical Corporation, Doha, Qatar; 3 Department of Emergency Medicine, Hamad Medical Corporation, Doha, Qatar; 4 Department of Nutrition Sciences, Qatar University, Doha, Qatar *Email: melhassan@qu.edu.qa

**Keywords:** cognitive memory performance, folate, cobalamin, Qatar Biobank, CANTAB

## Abstract

**Introduction:**

Cognitive health, crucial for the independence and quality of life in older adults, is influenced by various factors, including nutritional status, which is increasingly recognized for its importance. Folate (vitamin B9) and cobalamin (vitamin B12) are essential for neurological health. Despite most studies offering broad global insights, this research addresses the knowledge gap regarding the relationship between folate and cobalamin levels and cognitive memory performance in a cognitively healthy aging adult. The primary objective of this study is to examine the relationship between memory performance and blood levels of folate and cobalamin, as well as to identify the determinants of memory performance, in adults in Qatar.

**Methods:**

We conducted a cross-sectional analysis of the data obtained from Qatar Biobank. This study assessed cognitive performance using the Cambridge Neuropsychological Test Automated Battery and measured blood concentrations of folate and cobalamin. Additionally, we examined demographic, lifestyle, behavioral, and disease-related factors as determinants of memory performance. We used multivariable linear regression to identify associations between Paired Associated Learning First Attempt Memory (PALFAMS) and vitamin levels.

**Results:**

Six hundred and thirty-six individuals aged 40 years and older were included in this study. The z-scores for blood levels of folate and cobalamin were each found to be positively associated with the PALFAMS (β, 0.17 [95% CI, −0.188 to 0.538]; *P* = 0.334 and β, 0.19 [95% CI, −0.15 to 0.53]; *P* = 0.28, respectively), after adjustment for covariates. Older age and being male were found to have negative associations with PALFAMS (β, −0.10 [95% CI, −0.18 to −0.02]; *P* = 0.011 and β, −0.98 [95% CI, −1.91 to −0.05]; *P* = 0.040, respectively), whereas a higher level of education and the use of supplements showed positive associations with memory function (β, 3.76 [95% CI, 2.38 to 5.14]; *P* < 0.001 and β, 0.76 [95% CI, 0.02 to 1.50]; *P* = 0.044), after adjustment for covariates.

**Conclusion:**

Since the associations between blood levels of folate and cobalamin and memory performance were not statistically significant, these results underscore the need for more comprehensive studies to explore the complex relationships between nutrition and memory performance, ultimately guiding more effective strategies for the prevention and management of memory impairment.

## 1. INTRODUCTION

Cognition refers to the mental processes involved in interacting with people and the environment, ranging from visual perception to social understanding.^1^ The cognitive system is composed of six core functions: visuospatial skills, perceptual-motor abilities, learning and memory, executive function, language, and social cognition.^2^ Memory systems are structured to store specific types of information: factual memory for facts, episodic memory for events, and procedural memory for procedures.^2^ Cognitive abilities, including memory, develop from childhood through adulthood as the central nervous system matures. While aging can lead to a decline in some cognitive functions, factual memory, social cognition, and skills based on well-established information usually remain well-preserved in older age.^3^ Cognitive functions affected by aging include working memory, certain types of attention, episodic memory, and executive functions. The extent of cognitive decline varies among older adults; some experience significant declines, while others maintain strong cognitive performance.^4^ Major neurocognitive disorder, formerly known as dementia, is marked by a significant decline in one or more cognitive domains, representing a drop from previous cognitive levels that progressively worsens over time. It is also accompanied by a decline in the ability to perform daily activities.^5^

**Figure 1:**
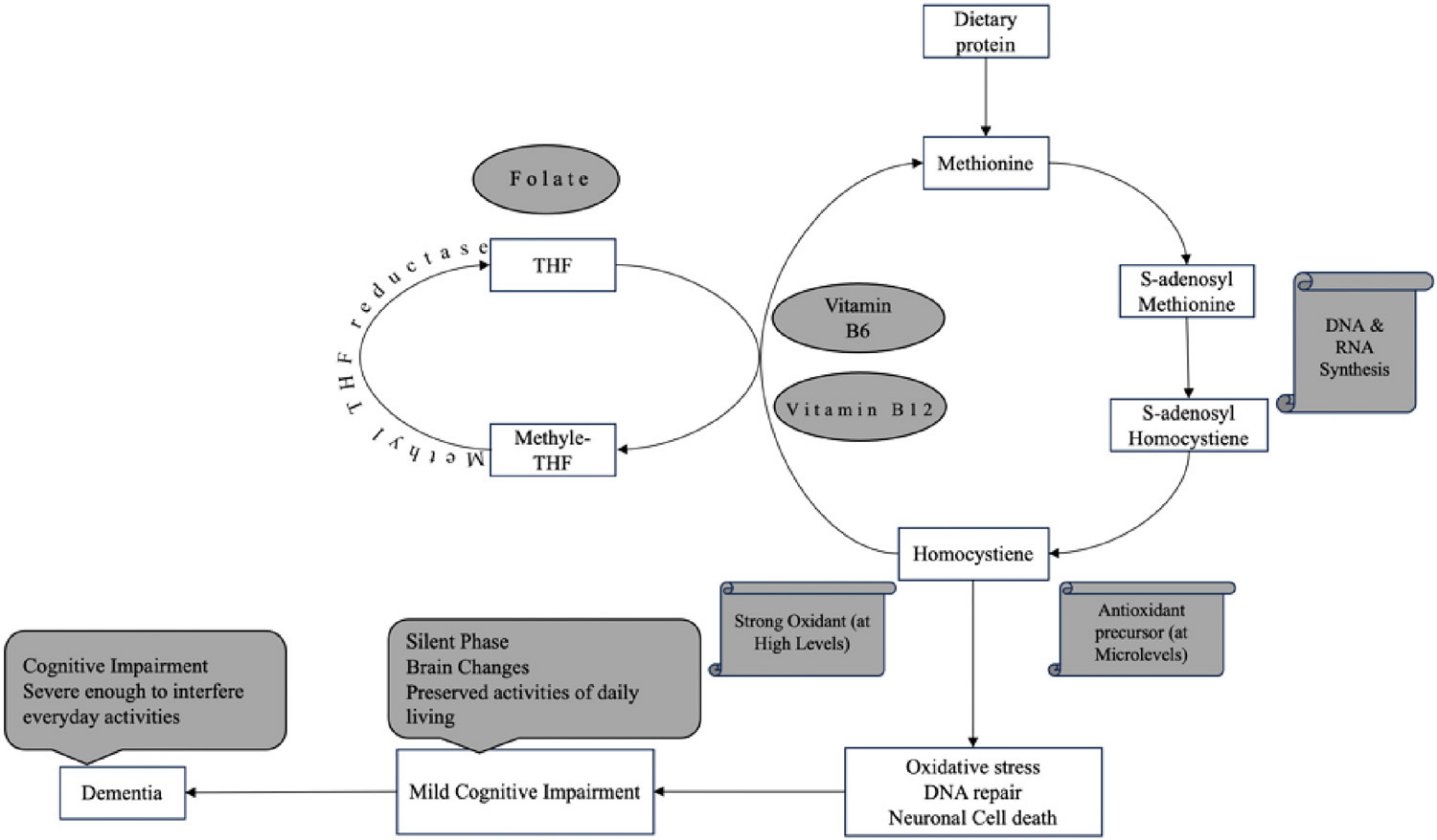
The role of folate and cobalamin in homocysteine metabolism and cognitive health. DNA: Deoxyribonucleic acid, RNA: Ribonucleic acid, THF: Tetrahydrofolate.

Cognitive health is vital for independence and quality of life in older adults.^1^ As the global population ages, understanding the factors affecting cognitive health is increasingly important.^6^ Regular physical activity, a nutritious diet, adequate sleep, and mental and social engagement help protect against cognitive decline,^7^ while smoking,^8^ excessive alcohol consumption,^9^ and poor sleep increase risk.^10^ Chronic conditions like diabetes, hypertension, and obesity further exacerbate this risk by inducing vascular and metabolic changes that affect brain health.^11^ Among these factors, nutritional status, particularly the levels of certain vitamins, has been identified as playing a crucial role in maintaining cognitive function, especially memory performance.^12^ Folate (vitamin B9), cobalamin (vitamin B12), and pyridoxine (vitamin B6) are crucial water-soluble vitamins for neurological function and cellular health.^13,14^ Folate is found in leafy greens and legumes,^13^ cobalamin in animal products like beef liver,^15^ and pyridoxine in fish, organ meats, and starchy vegetables.^16^ Deficiencies in folate and cobalamin can lead to cognitive impairment and, if not treated, ultimately dementia. These vitamins are key players in one-carbon metabolism, which is important for DNA synthesis, repair, and methylation (Figure 1).^17^ Given their crucial roles, it is hypothesized that adequate levels of folate and cobalamin may support cognitive memory performance and potentially mitigate age-related memory decline.

Approximately 55 million people worldwide were living with dementia in 2021, and this number is projected to rise to 78 million by 2030 and 152.8 million by 2050, with variations expected across different countries and regions.^18,19^ A study by Qassem et al. found that 1.33 million people in the Arab world had dementia in 2021, with prevalence rates in Qatar at 0.91% for those aged 50+ years and 2.84% for those aged 60+ years.^20^ Based on 2017 United Nations (UN) population estimates, it is conceivable that over 4400 individuals aged 60 years and above in Qatar may currently be grappling with dementia.^21^ Without effective prevention, treatment, or a cure, the number of individuals with memory impairment in Qatar could rise to 41,000 by 2050. Early screening, identifying treatable factors, and slowing disease progression are essential to prevent this increase.^21^ Addressing cognitive health globally is essential, considering regional challenges. Early identification of cognitive decline can improve the quality of life for aging individuals in Qatar, but research on cognitive health in the region is limited.

Our study aims to address this gap by examining the relationship between cognitive memory performance and blood levels of folate and cobalamin in healthy adults aged 40 and above, using the Cambridge Neuropsychological Test Automated Battery (CANTAB), which objectively measures various cognitive domains such as memory, attention, executive function, and decision-making.^22^ By conducting this study, we aim to understand how blood vitamin levels affect cognitive memory performance metrics, determine the factors influencing these changes, and identify the key determinants of memory performance, enhancing our understanding of the nutritional influences on memory in Qatar’s healthy aging population.

## 2. METHODS

This study involved secondary analysis of anonymized data from the Qatar Biobank (QBB), which collects extensive health-related information, including socio-demographics, health conditions, dietary habits, lifestyle, body composition, cognitive function, and biological specimens from up to 60,000 Qataris and long-term residents.^23^ A sample of 636 participants was selected for this study based on the inclusion and exclusion criteria. Inclusion criteria required participants to be aged 40 years and older and to have completed the CANTAB assessments. This age group is essential for the early detection and intervention of cognitive decline.^24^ Exclusion criteria included a history of neurological conditions, severe mental health disorders, or other conditions that could significantly impact cognitive function.

Cognitive function was assessed using the CANTAB test, specifically the Paired Associates Learning (PAL) test, which measures episodic memory, visual memory, and learning through pattern recall tasks on a screen.^22^ This study focused on the PAL First Attempt Memory Scores (PALFAMS), which indicate the number of correct box location identifications on the first attempt, excluding the 12-box level for standard comparison. Higher PALFAMS scores reflect better memory performance.^25^

The QBB measured blood levels of folate and cobalamin using standardized methods, providing continuous data. To account for variations in cutoff values from different instruments, the data were normalized using Z-scores, calculated as (x − mean)/SD. These standardized scores were then divided into tertiles for descriptive analysis. Vitamin B6 was excluded from the analysis because relevant laboratory data were not available in QBB during the study period.

The study used QBB data on socio-economic status, lifestyle habits, medical conditions, and dietary choices, collected through questionnaires on health, smoking, sociodemographics, sleep, physical activity, and diet. Nurse-administered interviews provided medical history, and about 60 mL of blood from each participant was collected for biomarker analysis.

Data categorization simplified variables for analysis: nationality (Qatari, non-Qatari), education level (primary or below, secondary, university or higher), employment status (unemployed, employed, retired), and sleep duration (short ≤7 hours, normal 7–8 hours, long >8 hours).^26^ Income was categorized into three ranges: ≤ 20,000 QAR, 20,001 to 50,000 QAR, and > 50,001 QAR per month. Smoking status was classified as non-smoker, ex-smoker, and current smoker.^27^ Physical activity was quantified in metabolic equivalents (METs)^28^ and categorized into no activity, low activity (METs < 10 excess), moderate activity (METs = 10–49.9 excess, and high activity (METs ≥ 50 excess).^28^ Body mass index (BMI) was categorized as underweight (BMI < 18.5), normal weight (BMI = 18.5–24.9), overweight (BMI = 25–29.9), and obese (BMI ≥ 30) based on World Health Organization (WHO) thresholds.^29^ Hypertension was defined by systolic blood pressure ≥140 mmHg, diastolic blood pressure ≥ 90 mmHg, or anti-hypertensive medication use.^30^ Diabetes was defined by self-reported diagnosis, glycosylated hemoglobin (HbA1c) level ≥ 6.5%, or random blood glucose > 11.1 mmol/L.^31^ Hypercholesterolemia was identified by a total serum cholesterol level of > 5 mmol/L or lipid-lowering medication use.^32^ Dietary intake and caffeine consumption were categorized as occasional or frequent, with diet profiles based on foods providing >20% of the daily value of folate and cobalamin.^33^ Homocysteine levels were included as a covariate in our analysis due to their interaction with folate and cobalamin and their impact on cognitive function. This accounts for multiple pathways affecting memory impairment, reduces confounding bias, and ensures a clearer understanding of the relationship between vitamin levels and cognitive memory performance.^34–36^

Statistical analysis was conducted using Stata 18.^37^ Descriptive statistics summarized participant characteristics, with continuous variables reported as means ± SD or medians and IQRs, and categorical data as frequencies and percentages. Linear regression models assessed the association between PALFAMS and folate and cobalamin z-scores, adjusting for confounders identified via literature review and DAG analysis. Model assumptions were validated through diagnostic tests, including CPR, kernel density, quantile-normal plots, and tests for normality, homoscedasticity, specification error, and multicollinearity using the Breusch-Pagan/Cook-Weisberg test, link test, and VIF analysis. Considering the fixed sample size of the study, we conducted a post hoc power analysis using Fisher’s z test for the correlation coefficient, assuming a small correlation between memory performance and blood levels of folate and cobalamin.^34^

## 3. RESULTS

The study included 636 participants with an average age of 52.4 years (SD = 8.3). The sample comprised 56% males,67.3% Qataris, with 59.9% holding a university degree or higher, and 70.7% employed. Among them, 44.2% were obese, 42.5% had no physical activity, and 68.8% slept 7 hours or less per night. Additionally, 24.4% had hypertension, 24.8% had diabetes, 56% had high cholesterol, and 4.6% had undergone bariatric surgery. The mean PALFAMS score was 10.25 (SD = 4.24; Table 1).

### 3.1 Cognitive memory performance metrics across folate and cobalamin Z-score tertiles

**Figure 2:**
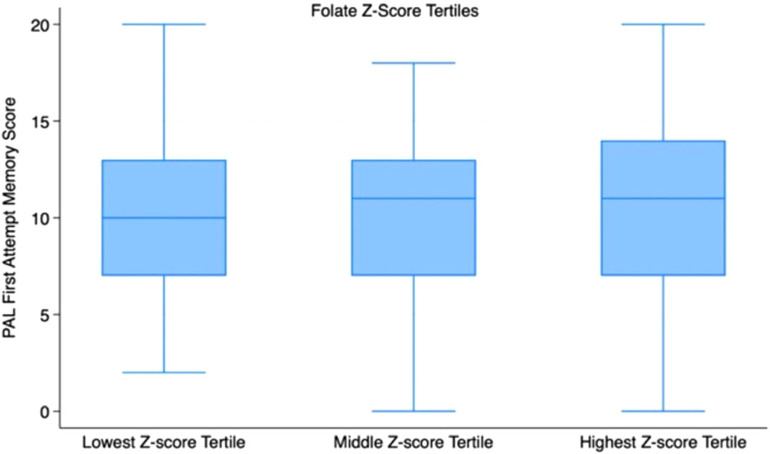
Boxplots of PALFAMS by folate Z-score tertiles.

**Figure 3:**
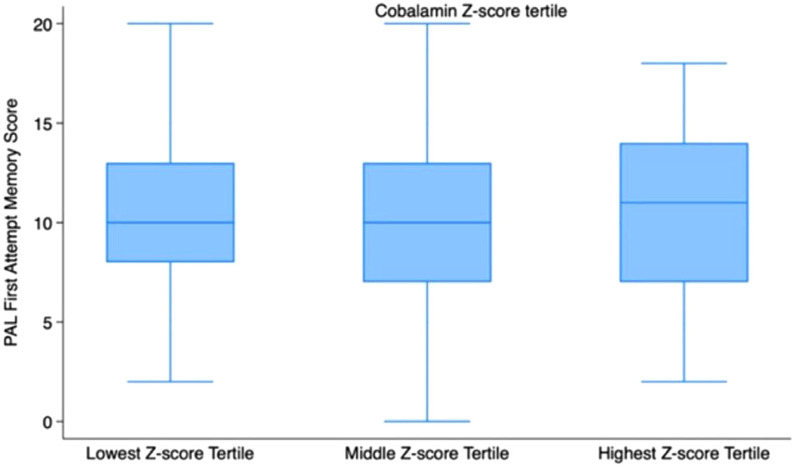
Boxplots of PALFAMS by cobalamin Z-score tertiles.

Figures 2 and 3 show that mean PALFAMS scores decrease in the lower tertiles of folate and cobalamin, suggesting a possible link between lower vitamin levels and reduced memory performance.

### 3.2 Association of folate and cobalamin with PALFAMS

**3.2.1 Folate and PALFAMS**

The initial unadjusted model showed a slight but non-significant positive association between folate and PALFAMS (β, 0.08 [95% CI, −0.26 to 0.41]; *P* = 0.65; Table 2). In the adjusted model, each standard deviation increase in folate was associated with a 0.17 increase in PALFAMS (β, 0.17 [95% CI, −0.19 to 0.54]; *P* = 0.344), accounting for various confounders and interactions. The adjusted model explained 22.7% of the variation in cognitive memory performance (R^2^, 0.2272; *P* < 0.001; Tables 2 and A1).

**3.2.2 Cobalamin and PALFAMS**

Similarly, the crude model showed a slight, non-significant association between cobalamin levels and PALFAMS (β, 0.14 [95% CI, −0.19 to 0.48]; *P* = 0.414; Table 2). In the adjusted model, a standard deviation increase in cobalamin levels was associated with a 0.19 increase in PALFAMS (β, 0.19 [95% CI, −0.15 to 0.53]; *P* = 0.28), after accounting for covariates. This adjusted model explained 21.4% of the variation in cognitive memory performance as measured by PALFAMS (R^2^, 0.2144; *P* < 0.001; Tables 2 and A2).

**3.2.3 Folate, Cobalamin, and PALFAMS**

The combined vitamin model, accounting for potential confounders, showed minimal change in beta coefficients compared to individual vitamin models but had better model fit (R^2^, 0.2246; *P* < 0.001; Tables 2 and 3).

### 3.3 Determinants of memory performance scores

**Figure 4:**
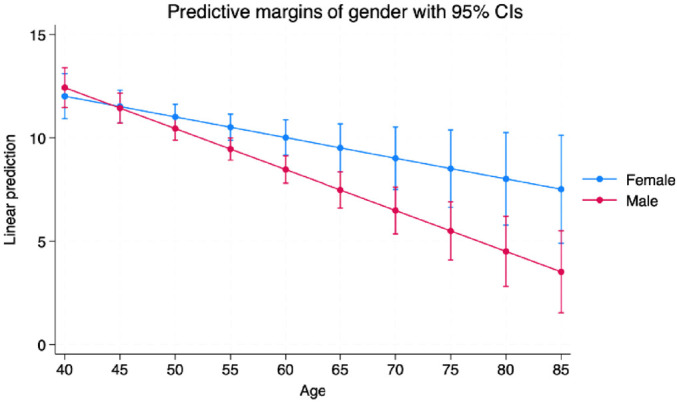
Margins plot demonstrating interaction between age and gender.

**Figure 5:**
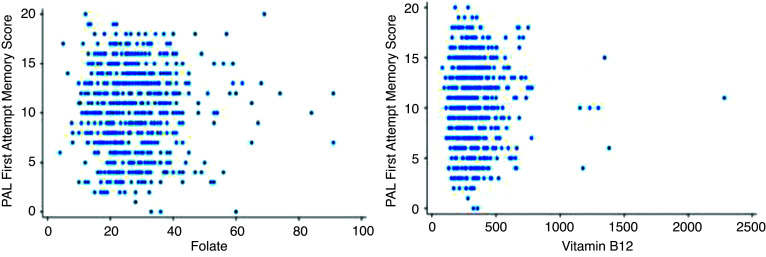
Scatterplots between cognitive function metrics and vitamin levels.

The analysis identified determinants of memory performance (Table 3), with age showing a significant negative association; each year over 40 corresponds to a 0.10 decline in PALFAMS (β, −0.10 [95% CI, −0.18 to −0.02], *P* = 0.011). A significant interaction between age and gender indicated a faster decline in memory performance in males compared to females, who tend to maintain higher cognitive performance, with greater variability in older adults of both genders (Figures 4 and 5).

Nationality was positively associated with memory performance, with Qataris exhibiting statistically significantly better memory scores as compared to non-Qataris by 0.91, after accounting for the confounders (β, 0.91 [95% CI, 0.10 to 1.72]; *P* = 0.027). Educational attainment was another strong predictor, with individuals holding secondary education (β, 2.44 [95% CI, 1.00 to 3.88], *P* = 0.001) and university or higher education (β, 3.76 [95% CI, 2.38 to 5.14]; *P* ≤ 0.001) demonstrating significantly better memory performance as compared to those with primary or no education, after adjusting for the confounders. Regular supplement use was associated with a 0.76 increase in PALFAMS, accounting for the potential covariates (β, 0.76 [95% CI, 0.02 to 1.50]; *P* = 0.044). No significant associations were found between memory performance and folate or cobalamin levels, physical activity, smoking, sleep, caffeine use, diet, homocysteine, high cholesterol, or bariatric surgery.

## 4. DISCUSSION

This study investigated the relationship between cognitive abilities and levels of folate and cobalamin in individuals aged 40 and above in Qatar’s healthy aging population. It focused on participants without diagnosed cognitive impairments, using a cross-sectional design with data from QBB. The study combined CANTAB memory assessments with biochemical analyses of folate and cobalamin to explore their correlation with memory performance, providing detailed insights into episodic and working memory.

Unlike previous research focused on individuals with cognitive impairments,^35^ our study uniquely investigates the role of folate and cobalamin in cognitive health within a cognitively healthy population, using the sensitive electronic CANTAB tests.^36^ This study aims to establish baseline vitamin correlations to detect early signs of memory decline. This approach, which departs from traditional cognitive assessments like the MMSE,^37^ MoCA,^38^ Alzheimer’s Disease Assessment Scale-Cognitive Subscale (ADAS-Cog),^39^ and Wechsler Memory Scale (WMS),^40^ seeks to provide new insights into the dietary role in cognitive reserve and memory maintenance in a healthy aging population.

Within the CANTAB framework, analyses focused on episodic memory using the PAL test and PALFAMS. Initial results showed a slight, non-significant positive association between higher folate levels and memory performance, aligning with Cheng et al., who found folate supplementation improved memory performance.^41^ Similarly, cobalamin levels showed a slight, non-significant positive association with PALFAMS, consistent with a multicenter RCT that linked improved memory performance to changes in cobalamin levels.^42^ However, other instruments were used to assess cognitive function, that is, the Rey Auditory Verbal Learning Test (RAVLT) and WMS, and assessed the interaction with the Mediterranean diet.^43^ The combined vitamin model supported findings by Ma et al., suggesting folate and cobalamin together enhance cognitive improvement more than individually.^44^ While no direct significant associations were found, variations in homocysteine levels across vitamin tertiles and their role in regression models indicate a complex relationship affecting cognitive abilities through biochemical pathways.^45^

Our investigation into cognitive memory performance highlights key determinants, including age, sex, nationality, education, and supplement use. Consistent with previous research, older age is associated with lower cognitive memory performance,^42,46^ while men outperform women in visual-episodic memory-related tasks.^46,47^ Qatari nationals show better memory performance, likely due to higher socioeconomic status, better healthcare, education, diet, and lower stress, aligning with studies on the role of race in memory performance. Higher education levels also correlate with improved memory, reflecting enhanced cognitive reserve.^45^ Yeung et al. identified supplement use as a key factor for cognitive reserve, supported by our study.^48^

Unlike previous research, our study did not find statistically significant associations between nutrient levels, lifestyle factors, and cognitive memory performance. Although folate and cobalamin deficiencies are known to lead to cognitive dysfunction and supplementation has been shown to improve cognitive function,^49,50^ these were not significant determinants in our study. Similarly, despite the known long-term effects of lifestyle factors like physical activity, smoking, and sleep patterns on memory function in aging individuals^51^ these factors were not significant determinants in our study.

The study, while comprehensive, acknowledges several limitations. Its cross-sectional design limits causal inferences due to a lack of temporality.^52^ Potential selection bias from QBB participants may affect generalizability to other populations, including within Qatar.^53^ The exclusive use of the CANTAB for cognitive assessment, without other tests like MMSE^37^ or MOCA,^38^ limits comparison across cognitive assessments. The lack of normative values for CANTAB in this population further restricts benchmarking. Missing data on pyridoxine also affects the understanding of overall vitamin status.^54^ Lastly, the sample size of 636, while practical, may limit the detection of subtle associations between vitamin levels and cognitive function. However, a retrospective power calculation using Fisher’s z test revealed that with this sample size, the study had approximately 71.5% power to detect a small difference in correlation coefficients of 0.1 with an alpha of 0.05, supporting the reliability of our findings.

Future studies should use longitudinal designs, incorporate multiple cognitive assessment tools, and aim for larger, more representative samples. Including normative values in cognitive testing and exploring genetic factors affecting nutrient metabolism could enhance understanding of how folate and cobalamin impact cognitive function.^7^ Addressing these areas will provide a clearer view of the relationships between vitamin levels and cognitive health.

## 5. CONCLUSION

This study explored the relationship between cognitive memory performance, as measured by PALFAMS, and blood levels of folate and cobalamin in Qatar’s healthy aging population. While no significant associations were found with these vitamins, age, gender, education, and nationality were key determinants of memory performance. The results suggest the need for further research on how nutrition and demographics affect cognitive aging to help prevent decline in older adults.

## DATA AVAILABILITY STATEMENT

The data supporting the findings of this study are not publicly available due to legal and ethical restrictions. Data access is restricted by Qatar Biobank’s policies to protect participant privacy and confidentiality. Researchers may request access to the data by contacting Qatar Biobank, subject to approval and compliance with their access guidelines.

## ETHICS APPROVAL

Ethical approval for this research was obtained through the Qatar Biobank data access application (QF-QBB-RES-ACC-00190). Informed consent was collected by Qatar Biobank from all participants as part of their standard data collection procedures. The Qatar University Institutional Review Board (QU-IRB) reviewed the project and determined that it is exempt from IRB review under exemption category #3 (reference number: QU-IRB 129/2024-EM).

## AUTHOR’S CONTRIBUTIONS

HA: Conception, design of the study, Data collection, analysis, and drafting the manuscript. MN: Supervision, revision of the manuscript critically for content. MAUR: Revision of the manuscript critically for content. ZS: Supervision, revision of the manuscript critically for content. MEA: Design of the study, analysis, supervision, and revision of the manuscript critically for content.

## FUNDING

This study was funded through the Qatar University Student Grant cycle 1, under approval number QUST-1-CHS-2024-1767.

## ACKNOWLEDGEMENTS

The authors would like to thank Qatar University for their support and resources, and all the faculty members and reviewers for their invaluable advice and encouragement during my Master of Public Health thesis. Special thanks to Qatar Biobank for providing the crucial data and assistance in overcoming challenges.

## CONFLICT OF INTEREST

The authors declare no conflicts of interest, financial or non-financial, related to this work. No personal, political, or professional influences have affected the preparation of this manuscript.

## Figures and Tables

**Table A1.** Linear regression: PALFAM versus Z-scores of folate (multivariable).

PALFAMS28	Coefficient (95% CI)	*P*-value*
zfol	0.18 (−0.19, 0.54)	0.344
can_age	−0.09 (−0.16, −0.01)	0.019
Male	4.63 (−0.23, 9.48)	0.062
Male × can_age	−0.10 (−0.19, −0.01)	0.025
Qatari	0.91 (0.12, 1.70)	0.024
Secondary education	2.57 (1.15, 3.99)	<0.001
University + education	3.73 (2.36, 5.09)	<0.001
Low activity: <10 excess	−0.07 (−1.09, 0.95)	0.895
Moderate activity: 10–<50 excess	−0.69 (−1.54, 0.16)	0.109
High activity: 50+ excess	−0.75 (−1.96, 0.45)	0.220
Ex-smoker	0.67 (−0.40, 1.74)	0.220
Current smoker	−0.29 (−1.34, 0.76)	0.588
Short sleep (≤7 hours)	1.06 (0.24, 1.88)	0.012
Long sleep (>8 hours)	0.19 (−1.23, 1.61)	0.790
Supplement: Yes	0.70 (−0.03, 1.43)	0.059
Frequent caffeine	0.57 (−0.75, 1.89)	0.396
Frequent diet folate	−0.88 (−1.98, 0.23)	0.120
Homocysteine	−0.03 (−0.09, 0.03)	0.272
Constant	11.55 (7.15, 15.95)	<0.001

*P-value significant at <0.05.

**Table A2.** Linear regression: PALFAM versus Z-scores of cobalamin (multivariable).

PALFAMS28	Coefficient (95% CI)	*P*-value*
Zb12	0.19 (−0.16, 0.54)	0.281
can_age	−1.07 (−1.82, −0.31)	0.006
Male	4.69 (−2.28, 9.60)	0.062
Male × can_age	−1.01 (−1.93, −0.10)	0.031
Qatari	0.69 (−0.11, 1.49)	0.092
Secondary education	2.24 (0.81, 3.66)	0.002
University + education	3.69 (2.33, 5.05)	<0.001
Low activity: <10 excess	−0.03 (−1.06, 1.00)	0.956
Moderate activity: 10–<50 excess	−0.78 (−1.64, 0.07)	0.071
High activity: 50+ excess	−0.91 (−2.13, 0.30)	0.141
Ex-smoker	0.82 (−0.27, 1.90)	0.136
Current smoker	−1.11 (−1.16, 0.94)	0.837
High cholesterol:Yes	0.38 (−0.32, 1.09)	0.287
Supplement: Yes	0.92 (0.19, 1.66)	0.014
Bariatric surgery: Yes	−0.80 (−2.48, 0.88)	0.349
Homocysteine	−0.02 (−0.098, 0.064)	0.679
Frequent diet B12	−0.59 (−1.45, 0.28)	0.183
Constant	13.00 (8.67, 17.33)	<0.001

*P-value significant at 0.05.

**Table 1.** Baseline characteristics of the study participants.

Descriptive characteristics	Summary statistics (*N* = 636)
Age, mean (SD)	52.4 (8.3)
Sex	
Female	280 (44.0)
Male	356 (56.0)
Nationality, *n* (%)	
Non-Qatari	208 (32.7)
Qatari	428 (67.3)
Highest level of education, *n* (%)	
Primary of the below	49 (7.9)
Secondary	201 (32.3)
University or higher	373 (59.9)
Employment status, *n* (%)	
Unemployed	98 (15.8)
Employed	440 (70.7)
Retired	84 (13.5)
Income per month, *n* (%)	
≤20,000 QAR per month	300 (48.6)
20,001–50,000 QAR per month	179 (29.0)
>50,001 QAR per month	138 (22.4)
BMI status, *n* (%)	
Normal weight	97 (15.3)
Overweight	258 (40.6)
Obese	281 (44.2)
MET (hours/week), *n* (%)	
No activity	270 (42.5)
Low activity: <10 excess	95 (14.9)
Moderate activity: 10–49.9 excess	200 (31.4)
High activity: ≥ 50 excess	71 (11.2)
Sleep duration, *n* (%)	
Short (≤7 hours)	428 (68.8)
Normal (7–8 hours)	148 (23.8)
Long (>8 hours)	46 (7.4)
Smoking status, *n* (%)	
Non-smoker	424 (66.7)
Ex-smoker	95 (14.9)
Current smoker	117 (18.4)
Hypertension, *n* (%)	
No	481 (75.6)
Yes	155 (24.4)
Diabetes, *n* (%)	
No	478 (75.2)
Yes	158 (24.8)
Hypercholesterolemia, *n* (%)	
No	280 (44.0)
Yes	356 (56.0)
History of bariatric surgery, *n* (%)	
No	607 (95.4)
Yes	29 (4.6)
PALFAMS, mean (SD)	10.25 (4.24)

Continuous variables are presented as mean (SD), and categorical variables are shown as frequency (percentages %).

BMI: Body mass index; MET: Metabolic equivalent; PALFAMS: Paired associated learning first attempt memory score.

**Table 2.** Comparative regression coefficients for different models assessing the effect of folate and cobalamin Z-scores on PALFAMS.

Model	Crude β (95% CI)	*P*-values^†^	Adjusted β (95% CI)	*P*-values^†^
PALFAMS with folate z-scores	0.08 (−0.26 to 0.42)	0.649	0.18 (−0.19, 0.54)*	0.344
PALFAMS with cobalamin z-scores	0.14(−0.2 to 0.48)	0.414	0.19 (−0.16, 0.54)**	0.281
PALFAMS with folate and cobalamin	-	-	Folate: 0.17 (−0.21, 0.55)	0.379
z-scores***			Cobalamin: 0.15 (−0.20, 0.50)	0.411

PALFAMS: Paired associated learning first attempt memory score; CI: Confidence interval.

*Adjusted for age, sex, nationality, education levels, physical activity score, smoking status, sleep duration, supplement use, Caffeine use, homocysteine level, diet rich in folate, interaction between age and sex.

**Adjusted for age, sex, nationality, education levels, physical activity score, hypercholesterolemia, smoking status, history of bariatric surgery, homocysteine level, and a diet rich in cobalamin, interaction between age and sex.

***Adjusted for age, sex, nationality, education levels, physical activity score, BMI, hypercholesterolemia, smoking status, history of bariatric surgery, sleep duration, caffeine use, supplement use, homocysteine level, and a diet rich in cobalamin and folate.

^†^*P*-values < 0.05 indicate statistical significance.

**Table 3.** Determinants of cognitive memory performance.

Memory score	Unadjusted coefficient (95% CI)	*P*-value*	Adjusted coefficient (95% CI)	*P*-value*
Folate level Z-scores	0.08 (−0.26 to 0.41)	0.649	0.17 (−0.21, 0.55)	0.379
Cobalamin level Z-scores	0.14 (−0.19 to 0.48)	0.414	0.15 (−0.20, 0.50)	0.411
Age	−0.19 (−0.22 to −0.15)	<0.001	−0.10 (−0.18, −0.02)	0.011
Sex				
Male	−0.41(−1.09 to 0.25)	0.222	4.33 (−0.66, 9.33)	0.089
Age × sex	-	-	−0.098 (−1.91, −0.05)	0.040
Nationality				
Qatari	0.40 (−0.31 to 1.10)	0.279	0.91 (0.10, 1.72)	0.027
Education				
Secondary education	2.68 (1.39 to 3.97)	<0.001	2.44 (1.00, 3.88)	0.001
University + education	4.00 (2.77 to 5.23)	<0.001	3.76 (2.38, 5.14)	0.001 <0.001
Physical activity (MET)				
Low activity: <10 excess	0.27 (−0.73 to 1.27)	0.594	−0.02 (−1.05, 1.02)	0.976
Moderate activity: 10–<50 excess	−0.54 (−1.32 to 0.24)	0.173	−0.75 (−1.61, 0.11)	0.090
High activity: 50+ excess	−0.27 (−1.39 to 0.84)	0.631	−0.94 (−2.17, 0.28)	0.131
Smoking				
Ex-smoker	0.89 (−0.57 to 1.84)	0.065	0.75 (−0.34, 1.84)	0.176
Current smoker	0.31 (−0.56 to 1.19)	0.480	−0.17 (−1.25, 0.90)	0.754
Sleep				
Normal sleep (7–8 hours)	−0.76 (−1.55 to 0.03)	0.061	−1.03 (−1.86, −0.19)	0.017
Long sleep (>8 hours)	−0.46 (−1.75 to 0.82)	0.483	−0.88 (−2.25, 0.48)	0.204
Supplement				
Yes	0.44 (−0.22 to 1.11)	0.191	0.76 (0.02, 1.50)	0.044
Frequent caffeine				
Yes	0.32 (−0.87 to 1.53)	0.591	0.82 (−0.55, 2.18)	0.239
Homocysteine	−0.10 (−0.17 to −0.04)	0.001	−0.01 (−0.09, 0.07)	0.766
High cholesterol				
Yes	0.15 (−0.52 to 0.82)	0.651	0.23 (−0.48, 0.95)	0.525
Bariatric surgery				
Yes	0.05 (−1.52 to 1.64)	0.945	−0.99 (−2.67, 0.70)	0.250
A frequent diet rich in cobalamin				
Yes	0.14 (−0.67 to 0.96)	0.727	−0.43 (−1.31, 0.45)	0.335
A frequent diet rich in folate				
Yes	−0.83 (−1.86 to 0.21)	0.118	−0.80 (−1.92, 0.34)	0.166

PALFAMS: Paired associated learning first attempt memory score; CI: Confidence interval.

*P-value significant at 0.05.
